# Electrophysiological evidence for abnormal glutamate-GABA association following psychosis onset

**DOI:** 10.1038/s41398-018-0261-0

**Published:** 2018-10-08

**Authors:** Daisuke Koshiyama, Kenji Kirihara, Mariko Tada, Tatsuya Nagai, Mao Fujioka, Eriko Ichikawa, Kazusa Ohta, Motoko Tani, Maiko Tsuchiya, Akiko Kanehara, Kentaro Morita, Kingo Sawada, Jun Matsuoka, Yoshihiro Satomura, Shinsuke Koike, Motomu Suga, Tsuyoshi Araki, Kiyoto Kasai

**Affiliations:** 1 Department of Neuropsychiatry, Graduate School of Medicine, The University of Tokyo, 7-3-1, Hongo, Bunkyo-ku, Tokyo 113-8655 Japan; 2 The International Research Center for Neurointelligence (WPI-IRCN) at The University of Tokyo Institutes for Advanced Study (UTIAS), The University of Tokyo, 7-3-1, Hongo, Bunkyo-ku, Tokyo 113-8655 Japan; 3 Department of Psychiatry, Kawamuro Memorial Hospital, 71, Kitashinbo, Joetsu-shi, Niigata 943-0109 Japan; 4 University of Tokyo Institute for Diversity & Adaptation of Human Mind (UTIDAHM), 3-8-1, Komaba, Meguro-ku, Tokyo 153-8902 Japan; 5 Center for Evolutionary Cognitive Sciences, Graduate School of Art and Sciences, The University of Tokyo, 3-8-1, Komaba, Meguro-ku, Tokyo 153-8902 Japan; 6 Department of Rehabilitation, Graduate School of Medicine, The University of Tokyo, 7-3-1, Hongo, Bunkyo-ku, Tokyo 113-8655 Japan

## Abstract

Previous studies have shown glutamatergic dysfunction and γ-aminobutyric acid (GABA)-ergic dysfunction in schizophrenia. Animal studies suggest that N-methyl-d-aspartate receptor (NMDAR) dysfunction and GABA-ergic dysfunction interact with each other and lead to alterations in excitatory/inhibitory balance. The NMDAR and GABAergic-interneuron functions may be indexed by mismatch negativity (MMN) and auditory steady-state gamma-band response (ASSR), respectively. However, no previous studies have tested the hypothesis of an abnormal association between MMN and gamma-band ASSR in the same patients to identify the in vivo evidence of NMDAR-GABA association during the early stages of psychosis. Participants were individuals with recent-onset schizophrenia (ROSZ; *N* = 21), ultra-high risk (UHR; *N* = 27), and healthy controls (HCs; *N* = 24). The MMN amplitude was significantly impaired in ROSZ (*p* = 0.001, *d* *=* 1.20) and UHR (*p* = 0.003, *d* *=* 1.01) compared with HCs. The intertrial phase coherence (ITC) index of gamma-band ASSR was significantly reduced in ROSZ compared with HCs (*p* < 0.001, *d* *=* *–*1.27) and UHR (*p* = 0.032, *d* *=* *–*0.75). The event-related spectral perturbation (ERSP) index of gamma-band ASSR was significantly smaller in ROSZ compared with HCs (*p* < 0.001, *d* = −1.21). The MMN amplitude was significantly correlated with the ITC in ROSZ (*r* = −0.69, *p* < 0.001). These findings provide the first in vivo evidence that an abnormal association of the electrophysiological indices of NMDAR and GABA dysfunctions may be present in recent-onset schizophrenia.

## Introduction

Glutamatergic dysfunction is considered to be one of the major hypotheses for the pathophysiology of schizophrenia^1–3^. In an initial clinical study, Krystal et al.^4^ showed that ketamine, an antagonist of N-methyl-d-aspartate receptors (NMDAR: a glutamate receptor), induces schizophrenia-like symptoms in healthy volunteers. Subsequent studies have shown abnormal metabolites of glutamatergic amino acids, which are co-agonists of NMDAR, in the plasma or cerebrospinal fluid (CSF)^5,6^, and in the anterior cingulate cortex (ACC) using proton magnetic resonance spectroscopy (^1^H-MRS)^7,8^ in patients with schizophrenia. In contrast, investigations on postmortem brains of patients with schizophrenia have revealed abnormalities in γ-aminobutyric acid (GABA)-ergic interneurons, such as a reduced expression of GABA-synthesizing enzyme glutamic acid decarboxylase 67 (GAD67) and parvalbumin (PV) in the cortical neurons^9–11^. Furthermore, clinical studies have shown a reduction of GABA in the ACC measured by ^1^H-MRS in chronic schizophrenia^12^ and in first-episode schizophrenia^13^.

Both glutamatergic dysfunction and GABAergic dysfunction may reflect an altered excitatory/inhibitory (E/I) balance in schizophrenia. Excitation is mediated by pyramidal neurons that release glutamate as neurotransmitters, and inhibition is mediated by interneurons that release GABA as neurotransmitters. Because pyramidal neurons and interneurons are connected with each other and make networks^14^, glutamatergic excitation and GABAergic inhibition affect each other and contribute to the E/I balance^15^. Recent animal studies have shown that interactions between abnormalities in NMDAR and dysfunction of GABAergic interneurons lead to alterations in E/I balance. Repeated administration of NMDAR antagonists^16–20^ and genetic deletion of NMDAR^21–23^ cause dysfunction of GABAergic interneurons that cause disinhibition of pyramidal neurons and lead to alterations in E/I balance. Decreased expression of the GABA synthetic enzyme GAD67 causes alterations in NMDAR-dependent processes and leads to alterations in E/I balance^24,25^. Therefore, glutamatergic dysfunction and GABAergic dysfunction in schizophrenia may interact with each other and lead to alterations in E/I balance.

The mismatch negativity (MMN), an electrophysiological index of auditory deviance detection, is expected to be a useful biomarker for NMDAR dysfunction in schizophrenia^26–29^, because NMDAR antagonists reduce MMN amplitude^30–32^. Many original investigations and subsequent meta-analyses have demonstrated MMN amplitude reduction in individuals with chronic schizophrenia^33–38^, first-episode psychosis^38–43^, and ultra-high risk for psychosis (UHR)^38,42,44–49^. Some previous studies have shown that MMN amplitude reduction predicts the onset of psychosis in individuals with UHR^47–49^. In those studies, the MMN in converters showed significantly greater amplitude reduction than that in non-converters. Furthermore, previous investigations have shown that in schizophrenia patients, MMN is correlated with the plasma level of glutamate^50^, which is significantly correlated with CSF levels of glutamate^51,52^, and with the level of glutamate in the ACC measured using ^1^H-MRS^53^.

In contrast, gamma-band auditory steady-state response (ASSR) is a candidate electrophysiological index of GABAergic abnormalities in the auditory cortex in schizophrenia. Cortical gamma oscillations are evoked by synaptic interactions between PV-positive GABAergic interneurons and pyramidal neurons^54,55^. Previous studies have reported deficits of gamma-band ASSR in chronic schizophrenia^56–62^ and first-episode psychosis^63–65^. For UHR, Tada et al.^65^ showed a specific time-course ASSR alteration, with reduction in the late-latency component, while leaving early-latency component intact. Recent meta-analysis confirmed impairment of the 40 Hz ASSR in patients with schizophrenia^66^.

Accordingly, we hypothesize that there is an abnormal association between NMDAR and GABAergic dysfunction surrounding the auditory cortex, which may lead to altered E/I balance at around the onset of schizophrenia. However, there is a lack of in vivo electrophysiological evidence of an association between NMDAR function and GABA function in individuals in the early stages of psychosis. In this study, we used MMN and gamma-band ASSR as sensitive electrophysiological markers of NMDAR and GABAergic interneurons, respectively. We predicted that impaired MMN and reduced gamma-band ASSR would show a correlation around the onset of schizophrenia.

## Materials and methods

### Subjects

We recorded electroencephalograms (EEGs) of 21 individuals with recent-onset schizophrenia (ROSZ), 27 individuals with UHR, and 24 healthy controls (HCs; Table 1). Fourteen of the participants had also participated in our previous MMN study^42^, and 38 had participated in our previous ASSR study^65^. Individuals with ROSZ or UHR were recruited from outpatient and inpatient units at the University of Tokyo Hospital. The HC group was recruited through advertisements at several universities in Tokyo. Inclusion criteria for ROSZ individuals were that they were diagnosed using the Diagnostic and Statistical Manual of Mental Disorders, Fourth Edition (DSM-IV), aged 15–40 years, and had onset of continuous psychotic symptoms within the past 60 months. Among a total of 21 ROSZ patient sample, *N* = 15 underwent EEG measurement as the biomarker assessments at Time 1 for the Integrative Neuroimaging Studies for Schizophrenia Targeting Early Intervention and Prevention (IN-STEP) project^67^ [all satisfied the criteria of first-episode schizophrenia (FES): continuous psychotic symptoms within the past 60 months and no history of antipsychotic drug treatment for more than 16 cumulative weeks at entry into the IN-STEP project]; *N* = 1 did not undergo EEG testing at Time 1, but instead underwent the initial EEG testing during the follow-up period of INSTEP; and *N* = 5 were newly recruited (*N* = 3 satisfied FES). Inclusion criteria for individuals with UHR were identified using the Structured Interview for Prodromal Symptoms (SIPS)^68^, and all were aged 15–30 years. Inclusion criteria of HC were that they were aged 15–40 years and had no personal history of psychiatric disease or a family history of axis I disorders in first-degree relatives. Exclusion criteria for all groups were neurological illness, traumatic brain injury with loss of consciousness for more than 5 min, history of electroconvulsive therapy, low premorbid intelligence quotient (IQ; below 70), previous alcohol/substance abuse or addiction, or a hearing impairment, which was assessed with a hearing test in both ears at 30-dB sound pressure level tone at 1000 Hz and 40-dB at 4000 Hz by audiometer. Written informed consent was obtained from each subject before participation. The Research Ethics Committee of the Faculty of Medicine at the University of Tokyo approved this study (approval No. 629 and 2226).

**Table 1:** Demographics of participants

	ROSZ	UHR	HC	Statistics
*N* (sex ratio M/F)^a^	21 (11/10)	27 (14/13)	24 (10/14)	*χ*^2^ = 0.696, *df* = 2, *p* = 0.706
Age (years)^b^	24.0 (6.7)	20.6 (3.9)	22.3 (3.0)	*F*_2,69_ = 3.04, *p* = 0.054
Education (year)^b^	13.4 (2.5)	13.1 (2.6)	14.5 (1.6)	*F*_2,69_ = 2.29, *p* = 0.109
Premorbid IQ^b^	104 (9.0)	105 (8.1)	110 (6.4)	*F*_2,69_ = 3.03, *p* = 0.055
DOI (weeks)	46.5 (47.0)			
PANSS^c^ total	68.9 (19.6)	67.7 (14.3)		*t*_46_ = 0.238, *p* = 0.813
Positive	15.2 (5.3)	14.3 (3.7)		*t*_46_ = 0.684, *p* = 0.497
Negative	18.8 (7.0)	17.3 (4.7)		*t*_46_ = 0.874, *p* = 0.387
General	34.9 (10.0)	36.1 (8.0)		*t*_46_ = –0.466, *p* = 0.643
GAF score^c^	38 (11)	48 (10)		*t*_46_ = –3.35, *p* = 0.002
Antipsychotic dose (mg/day)^c^	388 (284)	174 (262)		*t*_46_ = 2.70, *p* = 0.010

All values are shown as mean (standard deviation) except for the first row. Underlined results indicate *p* < 0.05

*ROSZ* recent-onset schizophrenia, *UHR* ultra-high risk, *HC* healthy control, *IQ* intelligence quotient, *DOI* duration of illness, *PANSS* positive and negative symptom scale, *GAF* global assessment of functioning, *ANOVA* analysis of variance, *df* degrees of freedom

^a^Chi-square test

^b^One-way ANOVA

^c^Independent *t*-test

The estimated premorbid IQ was assessed using the Japanese version of the National Adult Reading Test in all participants^69,70^. The Positive and Negative Syndrome Scale (PANSS)^71^ and Global Assessment of Functioning (GAF)^72,73^ were used for assessment of global clinical symptoms and functioning in all participants with ROSZ or UHR. Nineteen patients with ROSZ and 17 individuals with UHR took antipsychotic medication. The antipsychotic dose was converted to an equivalent dose of chlorpromazine^74^.

### Procedure and analyses of the mismatch negativity

A two-tone auditory oddball paradigm with 2000 stimuli was used for MMN. Standard tones (1000 Hz, 50 ms) were 90% of the stimuli and duration-deviant tones (1000 Hz, 100 ms) were 10% of the stimuli. All stimuli were 80 dB and had 1 ms rise/fall time. Stimulus onset asynchrony was 500 ms. While the participants watched a silent cartoon, the tones were presented binaurally through inserted earphones (Multi Trigger System, Medical Try System, Tokyo, Japan).

We used a 64-channel Geodesic EEG System (Electrical Geodesics Inc., Eugene, OR) to obtain EEG data. Electrodes were referenced to the vertex, and impedances were maintained below 50 kΩ. The sampling rate was 500 Hz. The analog filter bandpass was set at 0.1–100 Hz. We analyzed EEG data using EEGLAB^75^. The continuous EEG data were re-referenced to an average reference, digitally filtered at 0.1–20 Hz, and segmented from −100 to 500 ms relative to the stimulus onset. The mean of the pre-stimulus baseline was subtracted for baseline correction. Independent component analysis was used for eye blink correction. Epochs exceeding ± 100 μV at any electrode were rejected. After averaging across trials, the event-related potential (ERP) waveform in response to standard stimuli was subtracted from the ERP waveform in response to deviant stimuli.

The amplitude of MMN at seven electrodes around the FCz was used for MMN analysis because the largest MMN amplitudes were obtained with them (Supplementary Fig. 1). The MMN amplitude was measured using the mean voltage from 135 to 205 ms post stimuli, in accordance with previous studies^36,42,76^.

We performed another oddball paradigm with response to frequency deviants, and the oddball paradigms were counterbalanced. However, we did not employ the frequency-deviant MMN in subsequent analyses because the group difference of MMN was specific to the duration-deviant MMN.

### Procedure and analyses of the auditory steady-state response

The ASSR paradigm used in this study, which is described in detail elsewhere^65^, was similar to those used in previous studies from different laboratories^56,58^. Briefly, subjects were instructed to relax with their eyes open, and they received auditory stimuli presented binaurally through inserted earphones, the same as those used in the MMN session. We performed the ASSR session before the MMN session, within 30 min. We recorded ASSR at first to avoid muscle artifacts because gamma band ASSR is sensitive to muscle artifacts^77^. We measured both ASSR and MMN in total within 60 min. Click sounds (80 dB, 1 ms) presented in 500 ms trains at 20, 30, and 40 Hz served as the auditory stimuli. Click sound trains were 200 trains at each frequency. The intertrain interval was 500 ms. The sampling rate was 250 Hz. The analog filter bandpass was set at 0.1–100 Hz. We used 40 Hz ASSR data for subsequent analyses because impairment of 40 Hz ASSR in early stages of schizophrenia was confirmed in previous studies^63–65^.

We analyzed EEG data by using EEGLAB^75^. The continuous EEG data were re-referenced to an average reference, a high-pass filter (1 Hz) and a notch filter (50 Hz) were applied to them in order to remove artifacts, and they were segmented from −250 to 750 ms, relative to the stimulus onset. Independent component analysis was used for eye blink correction, and epochs exceeding ± 100 μV at any electrode were rejected.

We performed time-frequency analyses with a short-term Fourier transformation and then calculated intertrial phase coherence (ITC) and event-related spectral perturbation (ERSP) as indices of ASSR. The ITC indicates phase consistency across trials and ranges between 0 (random phase across trials) and 1 (identical phase across trials). The ERSP indicates event-related changes in power relative to a pre-stimulus baseline. Decreases in ITC and ERSP reflect reduced neural responses to auditory steady-state stimulation. We calculated the mean ITC and ERSP by averaging the data over stimulation time (0–500 ms) and stimulation frequency (40 Hz: 36–45 Hz). Because our previous study^65^ observed differential alteration of early- and late-time-course components of ITC and ERSP in early stages of psychosis, we additionally calculated the mean ITC and ERSP for each 100 ms-epoch for time-course analyses. We focused on the frontocentral electrode site (FCz) because the most prominent ASSR was found at FCz.

### Statistical analyses

We used SPSS (Version 23.0.0.0, IBM Corp., Armonk, NY, USA) for all statistical analyses. We employed *χ*^2^ tests, independent *t*-tests, and one-way ANOVAs for comparison of demographics and clinical characteristics among the groups. We performed one-way ANOVAs for comparison of the number of epochs and found no significant differences among groups [MMN, 161 ± 30 for ROSZ, 156 ± 33 for UHR, and 164 ± 24 for HCs (*F*_2, 69_ = 0.44, *p* = 0.65); ASSR, 182 ± 21 for ROSZ, 183 ± 16 for UHR, and 187 ± 10 for HCs (*F*_2, 69_ = 0.62, *p* = 0.54)]. For the main comparison of MMN amplitude, we used repeated measures ANOVA with the three groups as the between-subject factors and with seven electrodes around the FCz as the within-subject factor. For the main comparison of ITC (0–500 ms) and ERSP (0–500 ms), we used one-way ANOVA. As a supplementary analysis, we performed repeated measures ANOVA with the three groups as the between-subjects factor and with time blocks (0–100, 100–200, 200–300, 400–500 ms) as the within-subject factor. Greenhouse-Geisser correction was used for repeated measure ANOVAs when appropriate. In case of a significant group-by-time interaction, we used one-way ANOVA and post-hoc tests with Bonferroni correction for comparison of ITC and ERSP time blocks, and Cohen’s *d* effect sizes were calculated for group comparisons of MMN, ITC, and ERSP. The significance level was set at *p* < 0.05 (two-tailed).

The Pearson correlation coefficients (*r*) of MMN with ITC and ERSP in each group were calculated. *p* < 0.0014 (0.05/36) was considered statistically significant based on Bonferroni correction (two-tailed). Additionally, we performed correlation analyses between frequency-deviant MMN and ASSR [20, 30, and 40 Hz; ITC (0–500 ms) and ERSP (0–500 ms); *p* < 0.0028 (0.05/18) was considered statistically significant based on Bonferroni correction (two-tailed)] and between the duration-deviant MMN and ASSR [20 and 30 Hz; ITC (0–500 ms) and ERSP (0–500 ms); *p* < 0.0042 (0.05/12) was considered statistically significant based on Bonferroni correction (two-tailed)] in ROSZ, UHR and HCs for supplementary information (Supplementary Table 1 and 2, and Supplementary Text).

If we found a significant correlation of MMN with ITC and ERSP in the group, we further tested whether the correlation was specific to the group by comparing Fisher’s *r*-to-*z* transformed correlational coefficients between the groups. Potential effects of age and premorbid IQ on MMN and ITC were tested with correlation analyses between age and MMN/ITC, and between premorbid IQ and MMN/ITC in ROSZ because age (*F*_2, 69 = _3.04, *p* = 0.054; Table 1) and premorbid IQ (*F*_2, 69 = _3.03, *p* = 0.055; Table 1) were different among the three groups (ROSZ, UHR, and HC) at the trend level. Furthermore, we performed partial correlation analyses between MMN and ITC, after adjustment for age and premorbid IQ in ROSZ. In addition, we sought to parse out the effects of medication on our main findings. We first calculated Pearson’s *r* between the antipsychotic dose (chlorpromazine equivalents) and MMN/ITC in ROSZ. The partial correlation to adjust for medication effects was calculated for the combination of the MMN amplitude and ITC in ROSZ. The significance level was set at *p* < 0.05 (two-tailed).

## Results

### Mismatch negativity

**Fig. 1:**
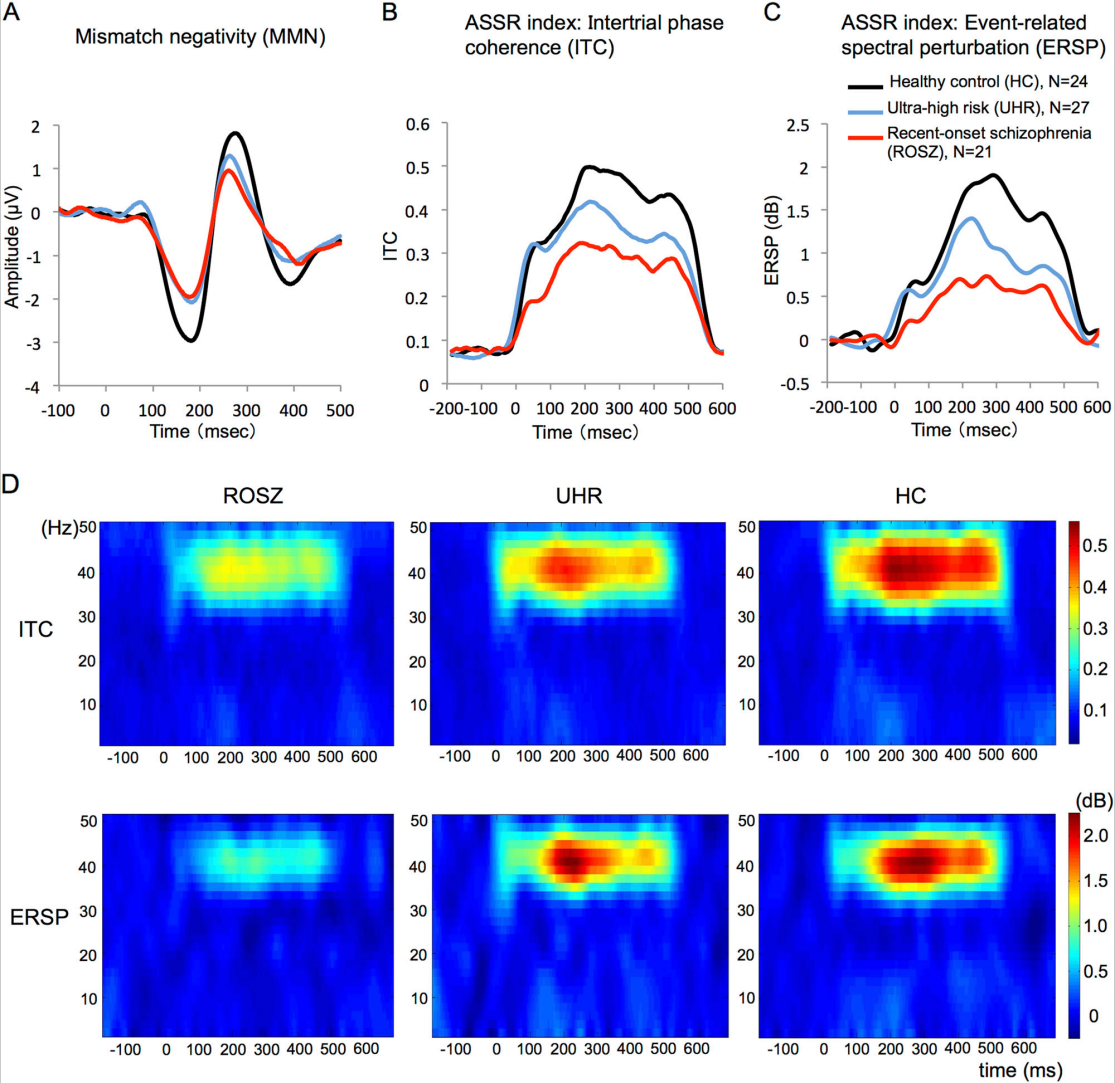
Mismatch negativity, intertrial phase coherence, and event-related spectral perturbation. The average waveforms of mismatch negativity (**a**), time-course of intertrial phase coherence (**b**), and event-related spectral perturbation (**c**) at the FCz. The red, blue, and black lines indicate data of participants in the recent-onset schizophrenia (ROSZ), ultra-high risk (UHR), and healthy control (HC) groups, respectively (**a**, **b**, and **c**). The *x*-axis indicates time (ms), and the *y*-axis indicates the amplitude of MMN (μV; **a**), and intertrial phase coherence (ITC; **b**) and event-related spectral perturbation (ERSP; dB; **c**) indices of auditory steady-state response (ASSR). The grand average time-frequency maps for ITC and ERSP at FCz (**d**). The *x*-axis indicates time (ms), *y*-axis indicates frequency (Hz), and color scale indicates intertrial phase coherence or event-related spectral perturbation at each time-frequency point (**d**)

The average waveforms for MMN in the ROSZ, UHR, and HC groups are shown in Fig. 1a. The mean amplitude (SD) of MMN was –1.48 (0.80) in the ROSZ group, –1.61 (0.84) in the UHR group, and –2.44 (0.81) in the HC group. Repeated measures ANOVA revealed a significant main effect of group (*F*_2, 67_ = 8.82, *p* < 0.001) and a significant main effect of electrode (*F*_4, 268_ = 10.9, *p* < 0.001) but no significant group-by-electrode interaction (*F*_8, 268_ = 0.29, *p* = 0.97). The post-hoc tests with Bonferroni correction indicated that the MMN amplitude was significantly smaller in the ROSZ group (*p* = 0.001, *d* = 1.20) and the UHR group (*p* = 0.003, *d* = 1.01) than in the HC group. There were no significant differences in MMN between ROSZ and UHR (*p* = 1.00, *d* = 0.16). Because we found no significant group-by-electrode interaction, we used the mean MMN amplitude at seven electrodes for further analyses.

### Auditory steady-state response

The time-courses of the ITC or ERSP, which are indices of ASSR, are shown in Fig. 1b, c. The grand average time-frequency maps for ITC or ERSP are shown in Fig. 1d. For the analysis of overall ITC (0–500 ms), there was a significant main effect of group (*F*_2, 69_ = 9.29, *p* < 0.001; Table 2). The post-hoc tests showed that ITC (0–500 ms) was significantly smaller in ROSZ than in UHR (*p* = 0.032, *d* = −0.75) and HCs (*p* < 0.001, *d* = −1.27). There were no significant differences in ITC (0–500 ms) between UHR and HCs (*p* = 0.20, *d* = −0.53). For additional analysis of time-course of ITC, repeated measures ANOVA showed a significant group-by-time interaction (*F*_5, 161_ = 5.41, *p* < 0.001). Subsequently, the post-hoc tests showed a significant difference between ROSZ and HCs in all five, between ROSZ and UHR in early latency (0–100, 100–200 ms), and between UHR and HCs in late latency (300–400, 400–500 ms) time blocks.

**Table 2:** Group comparison of ASSR indices (ITC and ERSP)

	One-way ANOVA							Post-hoc analysis^a^					
	ROSZ		UHR		HC		Statistics	ROSZ–UHR		ROSZ–HC		UHR–HC	
	Mean	SD	Mean	SD	Mean	SD		*p* ^a^	*d*	*p* ^a^	*d*	*p* ^a^	*d*
ITC [0–1, arbitrary unit]													
0–500 ms	0.27	0.12	0.35	0.11	0.41	0.11	*F*_2,69_ = 9.29, *p* < 0.001	0.032	–0.75	< 0.001	–1.27	0.202	–0.53
Time blocks													
0–100 ms	0.18	0.07	0.29	0.09	0.28	0.09	*F*_2,69_ = 12.6, *p* < 0.001	<0.001	–1.46	0.001	–1.23	1.000	0.20
100–200 ms	0.29	0.12	0.38	0.13	0.41	0.10	*F*_2,69_ = 5.86, *p* = 0.004	0.037	–0.70	0.005	–1.05	1.000	–0.24
200–300 ms	0.31	0.16	0.41	0.14	0.49	0.13	*F*_2,69_ = 8.66, *p* < 0.001	0.069	–0.64	< 0.001	–1.24	0.142	–0.60
300–400 ms	0.28	0.13	0.34	0.12	0.45	0.14	*F*_2,69_ = 9.27, *p* < 0.001	0.337	–0.48	< 0.001	–1.22	0.018	–0.79
400–500 ms	0.27	0.13	0.33	0.12	0.42	0.13	*F*_2,69_ = 8.32, *p* = 0.001	0.291	–0.50	< 0.001	–1.17	0.039	–0.72
ERSP [dB]													
0–500 ms	0.49	0.64	0.88	0.57	1.30	0.69	*F*_2,69_ = 9.18, *p* < 0.001	0.118	–0.64	< 0.001	–1.21	0.061	–0.67
Time blocks													
0–100 ms	0.17	0.41	0.51	0.45	0.50	0.41	*F*_2,69_ = 4.47, *p* = 0.015	0.026	–0.78	0.040	–0.79	1.000	0.03
100–200 ms	0.55	0.66	0.92	0.61	1.15	0.65	*F*_2,69_ = 4.99, *p* = 0.009	0.145	–0.59	0.007	–0.91	0.637	–0.36
200–300 ms	0.67	0.90	1.25	0.88	1.86	0.94	*F*_2,69_ = 9.67, *p* < 0.001	0.090	–0.65	< 0.001	–1.29	0.060	–0.67
300–400 ms	0.57	0.83	0.89	0.71	1.62	0.93	*F*_2,69_ = 9.84, *p* < 0.001	0.549	–0.42	< 0.001	–1.19	0.007	–0.88
400–500 ms	0.50	0.78	0.80	0.56	1.36	0.86	*F*_2,69_ = 8.13, *p* = 0.001	0.508	–0.44	0.001	–1.05	0.023	–0.78

*d* means Cohen’s *d* effect size. Underlined results indicate *p* < 0.05

*ANOVA* analysis of variance, *SD* standard deviation, *ROSZ* recent-onset schizophrenia, *UHR* ultra-high risk, *HC* healthy control, *ASSR* auditory steady-state response, *ITC* intertrial phase coherence, *ERSP* event-related spectral perturbation

^a^Bonferroni correction for three contrasts (ROSZ–UHR, ROSZ–HC, UHR–HC)

For the analysis of overall ERSP (0–500 ms), there was a significant main effect of group (*F*_2, 69_ = 9.18, *p* < 0.001; Table 2). The post-hoc tests showed that ERSP (0–500 ms) was significantly smaller in ROSZ than in HCs (*p* < 0.001, *d* = −1.21). There were no significant differences in ERSP (0–500 ms) between ROSZ and UHR (*p* = 0.12, *d* = −0.64) or between UHR and HCs (*p* = 0.061, *d* = −0.67). For additional analysis of the time-course of ERSP, a repeated measures ANOVA showed a significant group-by-time interaction (*F*_4, 151_ = 5.66, *p* < 0.001). The post-hoc tests showed a significant difference between ROSZ and HCs in all five, between ROSZ and UHR in early latency (0–100 ms), and between UHR and HCs in late latency (300–400, 400–500 ms) time blocks.

### Correlations between MMN amplitude and ASSR indices (ITC and ERSP)

**Fig. 2:**
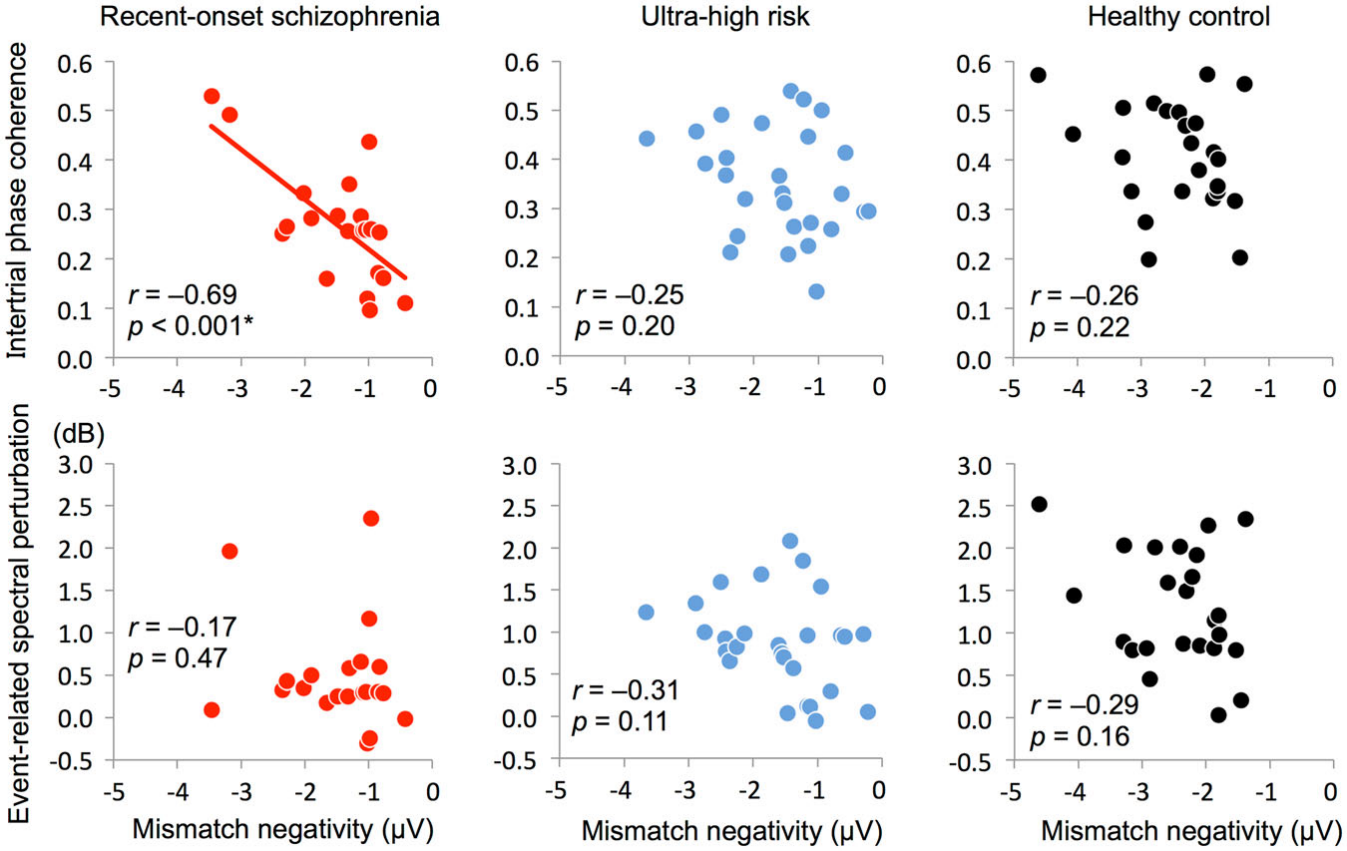
Correlation of mismatch negativity with intertrial phase coherence or event-related spectral perturbation. The *x*-axis indicates mismatch negativity (μV), and the *y*-axis indicates intertrial phase coherence or event-related spectral perturbation (dB). The asterisk indicates *p* < 0.0014 (0.05/36)

The MMN amplitude was significantly correlated with overall ITC (0–500 ms) in ROSZ (*r* = −0.69, *p* < 0.001; Table 3 and Fig. 2). However, the correlation between MMN amplitude and ITC (0–500 ms) was not significant in UHR (*r* = −0.25, *p* = 0.20) or HCs (*r* = −0.26, *p* = 0.22). The MMN amplitude was not significantly correlated with the overall ERSP (0–500 ms) for either group. Additionally, the MMN amplitude was not significantly correlated with ITC or ERSP for any time-course block.

**Table 3:** Correlation of MMN amplitude with ASSR indices (ITC and ERSP)

	MMN					
	ROSZ		UHR		HC	
	*r*	*p*	*r*	*p*	*r*	*p*
ITC						
0–500 ms	–0.69	< 0.001	–0.25	0.20	–0.26	0.22
Time blocks						
0–100 ms	–0.13	0.56	–0.04	0.86	–0.29	0.17
100–200 ms	–0.33	0.14	–0.26	0.19	–0.13	0.53
200–300 ms	–0.29	0.21	–0.33	0.10	–0.16	0.46
300–400 ms	–0.36	0.11	–0.27	0.17	–0.29	0.17
400–500 ms	–0.37	0.10	–0.18	0.38	–0.30	0.15
ERSP						
0–500 ms	–0.17	0.47	–0.31	0.11	–0.29	0.16
Time blocks						
0–100 ms	0.09	0.69	–0.19	0.34	–0.39	0.06
100–200 ms	–0.07	0.75	–0.17	0.40	–0.22	0.31
200–300 ms	–0.14	0.55	–0.32	0.10	–0.09	0.68
300–400 ms	–0.21	0.35	–0.36	0.07	–0.29	0.17
400–500 ms	–0.28	0.22	–0.29	0.14	–0.41	0.05

Underlined result indicates *p* < 0.0014 (0.05/36)

*ROSZ* recent-onset schizophrenia, *UHR* ultra-high risk, *HC* healthy control, *MMN* mismatch negativity, *ASSR* auditory steady-state response, *ITC* intertrial phase coherence, *ERSP* event-related spectral perturbation

The correlation between MMN and ITC was specific to the ROSZ group compared to the UHR group (*z* = 1.92, *p* = 0.055) and the HC group (*z* = 1.83, *p* = 0.067) at the trend level. Further, we evaluated potential effects of age and premorbid IQ on MMN and ITC with the correlation analyses between age and MMN/ITC, and between premorbid IQ and MMN/ITC in ROSZ. Age was not significantly correlated with MMN (*r* = 0.11, *p* = 0.64) or ITC (*r* = –0.23, *p* = 0.33) in ROSZ. Premorbid IQ was not significantly correlated with MMN (*r* = –0.22, *p* = 0.34) or ITC (*r* = 0.25, *p* = 0.28) in ROSZ. In addition, partial correlation analyses between MMN and ITC after adjustment for age (*r* = −0.69, *p* = 0.001) and premorbid IQ (*r* = −0.68, *p* = 0.001) showed almost the same results compared with the original results in ROSZ. An antipsychotic dose was not significantly correlated with MMN (*r* = 0.29, *p* = 0.21) or ITC (0–500 ms; *r* = −0.42, *p* = 0.061) in ROSZ. A partial correlational analysis after adjustment for the antipsychotic dose between MMN and ITC (0–500 ms) in ROSZ was performed, and the correlation remained significant (*r* = −0.66, *p* = 0.002).

## Discussion

We obtained the following results: (i) the MMN amplitude was reduced in ROSZ and UHR; (ii) ITC and ERSP were significantly reduced in ROSZ, and early-latency ITC and ERSP were intact, while late-latency ITC and ERSP were impaired in UHR; and (iii) as the main finding, the MMN amplitude was significantly correlated with the ITC in ROSZ, but not in UHR. This study is the first to identify an association between electrophysiological indices of glutamate and GABA function in vivo in early psychosis.

Our results of reduced MMN both in ROSZ and UHR are consistent with those of previous studies on early stages of psychosis^39–47^. These findings indicate that NMDAR shows dysfunction before the onset of schizophrenia. The overall indices of gamma-band ASSR (ITC and ERSP) are also reduced in recent-onset schizophrenia, consistent with previous findings^63,65^. Confirming our previous investigation^65^, in UHR, early-latency ITC and ERSP were intact, while late-latency ITC and ERSP were impaired. These patterns of abnormality suggest that GABAergic interneuron dysfunction may, at least in part, develop through the onset of psychosis, which might explain the observation that an abnormal association of MMN and ASSR is evident in ROSZ alone.

The main finding of our study was a significant correlation between the MMN amplitude and gamma-band ASSR in ROSZ, but not in UHR. The correlation was specific to the ROSZ group compared to the UHR group at the trend level. This may reflect altered E/I balance in ROSZ but not in UHR. Previous animal studies have shown that NMDAR hypofunction causes dysfunction of GABAergic interneurons^21–23^. Belforte et al^21^. found that elimination of the NR1 subunit of the NMDAR in cortical and hippocampal interneurons caused a reduction in the expression of GAD67 and PV. Carlen et al.^23^ reported that NMDAR dysfunction in PV interneurons caused impairments in gamma oscillations. Furthermore, Nakazawa et al.^78^ described that the first NMDAR hypofunction occurs in PV-positive GABA interneurons, in early postnatal development, which would impair the cortical maturation that causes a reduction in intrinsic excitability and impaired GABA release, thus leading to the disinhibition of pyramidal neurons. Altogether, these findings suggest that the dysfunction of NMDARs cause dysfunction of GABA interneurons that cause disinhibition of pyramidal neurons and leads to alterations in E/I balance. Decreased expression of the GABA synthetic enzyme GAD67 causes alterations in NMDAR-dependent processes and lead to alterations in E/I balance^24,25^. Therefore, glutamatergic dysfunction and GABAergic dysfunction may interact with each other and lead to altered E/I balance in ROSZ. On the other hand, individuals with UHR showed reduced MMN and ASSR, but there were no significant correlations between MMN and ASSR. These findings suggest that individuals with UHR may have glutamatergic dysfunction and GABAergic dysfunction but that interactions between glutamatergic dysfunction and GABAergic dysfunction may not be strong enough to lead to an altered E/I balance in UHR. Therefore, glutamatergic dysfunction and GABAergic dysfunction may indicate a risk for psychosis, and interactions between glutamatergic dysfunction and GABAergic dysfunction and subsequent alterations in E/I balance may lead to the onset of psychosis. In contrast to ITC, ERSP showed no significant correlation with MMN. Previous animal studies have shown that dysfunction of NMDAR on GABAergic interneuron decrease evoked power of gamma oscillations but increase spontaneous power of gamma oscillations^23,79^. Because ERSP reflects power of gamma oscillations and includes both evoked power and spontaneous power, the mixed effects of decreased evoked power and increased spontaneous power may obscure the association between MMN and gamma oscillations. Because ITC does not reflect the power of gamma oscillations, the mixed effects of power do not affect the association.

The study has some limitations. First, potential medication effects might have influenced our findings. However, our main findings remained significant when we applied a partial correlation analysis to adjust for medication effects. Second, this study was a cross-sectional study. Future longitudinal studies will be required to clarify the longitudinal course of the association between MMN amplitude and gamma-band ASSR around the onset of psychosis.

In conclusion, our observations provide the first in vivo electrophysiological evidence that an abnormal association of NMDAR-GABA dysfunctions presumably surrounding the auditory cortex may be present in recent-onset schizophrenia. The MMN and gamma-band ASSR may be useful sensitive markers in the development of early intervention strategies for psychosis, in order to target the alleviation of this aberrant association.

## Electronic supplementary material

Supplementary text

Supplementary fig1

Supplementary fig legends

Supplementary tables
