# Supplementary material for: Electrophysiological evidence for abnormal glutamate-GABA association following psychosis onset
Source: Transl Psychiatry. 2018 Oct 8;8:211. doi: 10.1038/s41398-018-0261-0 (PMC6175929; doi:10.1038/s41398-018-0261-0)
Supplement: Supplementary file 1 — Supplementary text [file 41398_2018_261_MOESM1_ESM.docx]

**Supplementary Text**

**Electrophysiological evidence for abnormal glutamate-GABA association following psychosis onset**

Daisuke Koshiyama, M.D.^1^; Kenji Kirihara, M.D., Ph.D.^1^; Mariko Tada, M.D., Ph.D.^1, 2^; Tatsuya Nagai, M.D., Ph.D.^1, 3^; Mao Fujioka, M.D.^1^; Eriko Ichikawa, M.A.^1^; Kazusa Ohta, Ph.D.^1^; Motoko Tani, M.A.^1^; Maiko Tsuchiya, M.A.^1^; Akiko Kanehara, M.P.H^1^; Kentaro Morita, M.D.^1^; Kingo Sawada, M.D.^1^; Jun Matsuoka, M.D.^1^; Yoshihiro Satomura, M.D., Ph.D.^1^; Shinsuke Koike, M.D., Ph.D.^1, 2, 4, 5^; Motomu Suga, M.D., Ph.D.^1, 6^; Tsuyoshi Araki, M.D., Ph.D.^1^; Kiyoto Kasai M.D., Ph.D.^1, 2^

1. Department of Neuropsychiatry, Graduate School of Medicine, The University of Tokyo, 7-3-1, Hongo, Bunkyo-ku, Tokyo 113-8655, Japan
2. The International Research Center for Neurointelligence (WPI-IRCN) at The University of Tokyo Institutes for Advanced Study (UTIAS), The University of Tokyo, 7-3-1, Hongo, Bunkyo-ku, Tokyo 113-8655, Japan
3. Department of Psychiatry, Kawamuro Memorial Hospital, 71, Kitashinbo, Joetsu-shi, Niigata 943-0109, Japan
4. University of Tokyo Institute for Diversity & Adaptation of Human Mind (UTIDAHM), 3-8-1, Komaba, Meguro-ku, Tokyo 153-8902, Japan
5. Center for Evolutionary Cognitive Sciences, Graduate School of Art and Sciences, The University of Tokyo, 3-8-1, Komaba, Meguro-ku, Tokyo 153-8902, Japan
6. Department of Rehabilitation, Graduate School of Medicine, The University of Tokyo, 7-3-1, Hongo, Bunkyo-ku, Tokyo 113-8655, Japan

**TABLE OF CONTENTS**

Supplementary Results………………………....…………...……...3

Supplementary Discussion…………………..……………...……...3

Supplementary References………………………….……………...5**Supplementary Results**

For supplementary information, the results of correlations between frequency-deviant MMN and ASSR [20, 30, and 40 Hz; ITC (0–500 ms) and ERSP (0–500 ms)] and between duration-deviant MMN and ASSR [20 and 30 Hz; ITC (0–500 ms) and ERSP (0–500 ms)] in ROSZ, UHR and HCs are shown in **Supplementary Table 1 and 2**. We did not find any significant correlations between frequency-deviant MMN and ASSR (20, 30, and 40 Hz) in ROSZ, UHR or HCs. On the other hand, we found a significant correlation between duration-deviant MMN and ITC index of 20 Hz ASSR (*r* = –0.61, *p* = 0.003). Additionally, duration-deviant MMN was correlated with an ITC index of 30 Hz ASSR (*r* = –0.44, *p* = 0.048) at an uncorrected significant level (*p* < 0.05).

**Supplementary Discussion**

Any significant correlations between frequency-deviant MMN and ASSR (20, 30, and 40 Hz) were not found in ROSZ, UHR or HCs. In addition to a lack of a significant difference in frequency-deviant MMN amplitude among the three groups, these results may be due to a low signal-to-noise ratio in frequency-deviant MMN compared to those in duration-deviant MMN.^1^

We found correlations between duration-deviant MMN and an ITC index of 20 Hz ASSR (*r* = –0.61, *p* = 0.003) and that of 30 Hz ASSR (*r* = –0.44, *p* = 0.048). These correlations may be due to the fact that 20 Hz and 30 Hz ASSR also reflect GABAergic interneuron function.^2^ However, 40 Hz ASSR is the largest among 20 Hz, 30 Hz and 40 Hz ASSR in human scalp EEG.^3, 4^ Furthermore, previous studies could not detect differences between patients with schizophrenia and healthy controls using 20 Hz and 30 Hz ASSR due to the worse signal-to-noise ratio ^5-9^. Thus, we focused on 40 Hz ASSR in this study.**Supplementary References**

1. Avissar M, Xie S, Vail B, Lopez-Calderon J, Wang Y, Javitt DC. Meta-analysis of mismatch negativity to simple versus complex deviants in schizophrenia. *Schizophr Res* 2018; **191:** 25-34.

2. Cardin JA, Carlen M, Meletis K, Knoblich U, Zhang F, Deisseroth K *et al.* Driving fast-spiking cells induces gamma rhythm and controls sensory responses. *Nature* 2009; **459:** 663-667.

3. Artieda J, Valencia M, Alegre M, Olaziregi O, Urrestarazu E, Iriarte J. Potentials evoked by chirp-modulated tones: a new technique to evaluate oscillatory activity in the auditory pathway. *Clin Neurophysiol* 2004; **115:** 699-709.

4. Galambos R, Makeig S, Talmachoff PJ. A 40-Hz auditory potential recorded from the human scalp. *Proc Natl Acad Sci U S A* 1981; **78:** 2643-2647.

5. Kwon JS, O'Donnell BF, Wallenstein GV, Greene RW, Hirayasu Y, Nestor PG *et al.* Gamma frequency-range abnormalities to auditory stimulation in schizophrenia. *Arch Gen Psychiatry* 1999; **56:** 1001-1005.

6. Light GA, Hsu JL, Hsieh MH, Meyer-Gomes K, Sprock J, Swerdlow NR *et al.* Gamma band oscillations reveal neural network cortical coherence dysfunction in schizophrenia patients. *Biol Psychiatry* 2006; **60:** 1231-1240.

7. Tsuchimoto R, Kanba S, Hirano S, Oribe N, Ueno T, Hirano Y *et al.* Reduced high and low frequency gamma synchronization in patients with chronic schizophrenia. *Schizophr Res* 2011; **133:** 99-105.

8. Spencer KM, Salisbury DF, Shenton ME, McCarley RW. Gamma-band auditory steady-state responses are impaired in first episode psychosis. *Biol Psychiatry* 2008; **64:** 369-375.

9. Tada M, Nagai T, Kirihara K, Koike S, Suga M, Araki T *et al.* Differential alterations of auditory gamma oscillatory responses between pre-onset high-risk individuals and first-episode schizophrenia. *Cereb Cortex* 2016; **26:** 1027-1035.
