# Supplementary figures and images for: Electrophysiological evidence for abnormal glutamate-GABA association following psychosis onset
Source: Transl Psychiatry. 2018 Oct 8;8:211. doi: 10.1038/s41398-018-0261-0 (PMC6175929; doi:10.1038/s41398-018-0261-0)

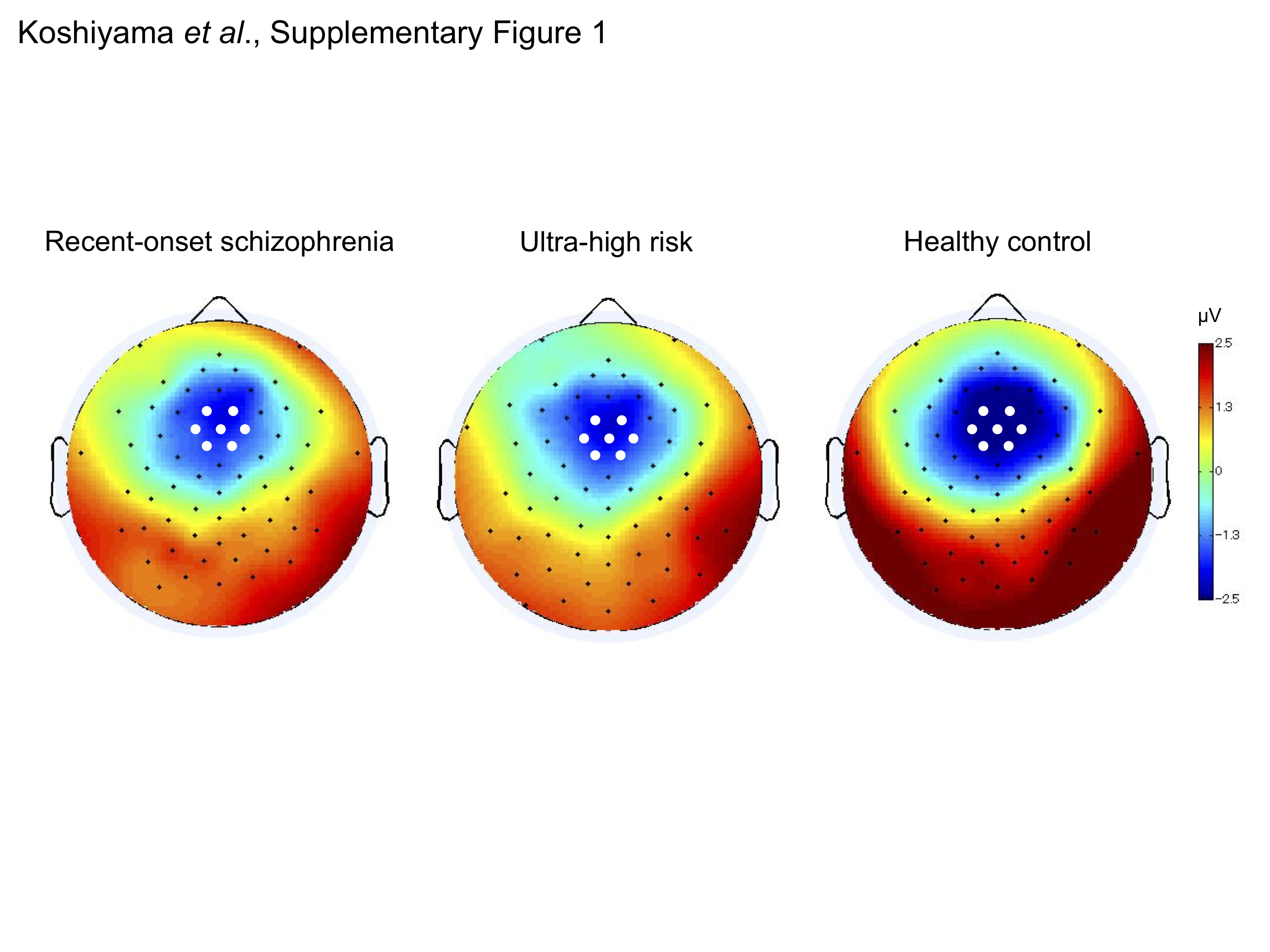

Supplement: Supplementary file 2 — Supplementary fig1 [file 41398_2018_261_MOESM2_ESM.tif]
