# Supplementary material for: Electrophysiological evidence for abnormal glutamate-GABA association following psychosis onset
Source: Transl Psychiatry. 2018 Oct 8;8:211. doi: 10.1038/s41398-018-0261-0 (PMC6175929; doi:10.1038/s41398-018-0261-0)
Supplement: Supplementary file 3 — Supplementary fig legends [file 41398_2018_261_MOESM3_ESM.docx]

**Supplementary figure legends**

**Supplementary Figure 1** Topographies for MMN amplitude in recent-onset schizophrenia, ultra-high risk, and healthy control groups.

Legends: The mean amplitude of MMN at seven electrodes around the FCz (white circles) was used for MMN analysis because the largest MMN amplitudes were obtained with them.

Abbreviations: MMN, mismatch negativity.
