# Supplementary material for: Electrophysiological evidence for abnormal glutamate-GABA association following psychosis onset
Source: Transl Psychiatry. 2018 Oct 8;8:211. doi: 10.1038/s41398-018-0261-0 (PMC6175929; doi:10.1038/s41398-018-0261-0)
Supplement: Supplementary file 4 — Supplementary tables [file 41398_2018_261_MOESM4_ESM.docx]

**Supplementary Table 1** Correlation of frequency MMN amplitude with 20 Hz, 30 Hz, and 40 Hz ASSR indices [ITC (0–500 ms) and ERSP (0–500 ms)]

|  | Frequency MMN | | | | | |
| --- | --- | --- | --- | --- | --- | --- |
|  | ROSZ | | UHR | | HC | |
|  | *r* | *p* | *r* | *p* | *r* | *p* |
| 20 Hz ASSR |  |  |  |  |  |  |
| ITC | –0.22 | 0.33 | 0.03 | 0.90 | –0.09 | 0.67 |
| ERSP | –0.09 | 0.70 | –0.26 | 0.19 | <0.01 | 1.00 |
| 30 Hz ASSR |  |  |  |  |  |  |
| ITC | –0.14 | 0.54 | –0.15 | 0.46 | 0.10 | 0.63 |
| ERSP | 0.28 | 0.23 | –0.26 | 0.19 | 0.27 | 0.19 |
| 40 Hz ASSR |  |  |  |  |  |  |
| ITC | –0.15 | 0.53 | –0.32 | 0.11 | –0.15 | 0.48 |
| ERSP | –0.02 | 0.95 | –0.35 | 0.07 | –0.11 | 0.60 |

Legend: Significant level was set at *p* < 0.0028 (0.05/18).

Abbreviations: ROSZ, recent-onset schizophrenia; UHR, ultra-high risk; HC, healthy control; MMN, mismatch negativity; ASSR, auditory steady state response; ITC, intertrial phase coherence; ERSP, event-related spectral perturbation.

**Supplementary Table 2** Correlation of duration MMN amplitude with 20 Hz and 30 Hz ASSR indices [ITC (0–500 ms) and ERSP (0–500 ms)]

|  | Duration MMN | | | | | |
| --- | --- | --- | --- | --- | --- | --- |
|  | ROSZ | | UHR | | HC | |
|  | *r* | *p* | *r* | *p* | *r* | *p* |
| 20 Hz ASSR |  |  |  |  |  |  |
| ITC | –0.61 | 0.003 | –0.06 | 0.77 | –0.15 | 0.48 |
| ERSP | –0.38 | 0.09 | –0.17 | 0.41 | –0.17 | 0.42 |
| 30 Hz ASSR |  |  |  |  |  |  |
| ITC | –0.44 | 0.048 | –0.15 | 0.46 | <0.01 | 0.99 |
| ERSP | 0.03 | 0.91 | –0.24 | 0.23 | 0.19 | 0.36 |

Legend: Underlined result indicates *p* < 0.0042 (0.05/12).

Abbreviations: ROSZ, recent-onset schizophrenia; UHR, ultra-high risk; HC, healthy control; MMN, mismatch negativity; ASSR, auditory steady state response; ITC, intertrial phase coherence; ERSP, event-related spectral perturbation.
